# Exploring the utility of alcohol flushing as an instrumental variable for alcohol intake in Koreans

**DOI:** 10.1038/s41598-017-18856-z

**Published:** 2018-01-11

**Authors:** Yoonsu Cho, Soyoung Kwak, Sarah J. Lewis, Kaitlin H. Wade, Caroline L. Relton, George Davey Smith, Min-Jeong Shin

**Affiliations:** 1 Department of Public Health Sciences, BK21PLUS Program in Embodiment: Health-Society Interaction, Graduate School, Korea University, Seoul, Republic of Korea; 2 MRC Integrative Epidemiology Unit, Population Health Sciences, Bristol Medical School, University of Bristol, Bristol, UK

## Abstract

Previous studies have indicated an association of higher alcohol intake with cardiovascular disease and related traits, but causation has not been definitively established. In this study, the causal effect of alcohol intake on hypertension in 2,011 men and women from the Ansan-Ansung cohort was estimated using an instrumental variable (IV) approach, with both a phenotypic and genotypic instrument for alcohol intake: alcohol flushing and the rs671 genotype (in the alcohol dehydrogenase 2 [*ALDH2*] gene), respectively. Both alcohol flushing and the rs671 genotype were associated with alcohol intake (difference in alcohol intake with alcohol flushers vs. non-flushers: −9.07 g/day; 95% confidence interval [CI]: −11.12, −7.02; P-value: 8.3 × 10^−18^ and with the rs671 GA + AA vs. GG genotype: −7.94 g/day; 95% CI: −10.20, −5.69; P-value: 6.1 × 10^−12^). An increase in alcohol intake, as predicted by both the absence of alcohol flushing and the presence of the rs671 GG genotype in the IV analyses, was associated with an increase in blood pressure in men from this Korean population. In conclusion, this study supports a causal effect of alcohol intake on hypertension and indicated that alcohol flushing may be a valid proxy for the *ALDH2* rs671 polymorphism, which influences alcohol intake in this Korean population.

## Introduction

Alcohol ranks sixth among factors that increase the risk of disease-related mortality and disability^1^. Previous studies have revealed an association of alcohol consumption and cardiovascular disease (CVD) and related traits, including blood pressure (BP) and hypertension, but causation has not been definitively established. Additionally, some observational studies have reported J-shaped curves in the relationship between alcohol use and CVD, where light-to-moderate alcohol use is cardio-protective^2,3^. However, observational studies are subject to bias due to confounding and reverse causality. For example, elevated cardiovascular risk observed in non-drinkers could be explained by individuals refraining from alcohol drinking because of their poor health status (reverse causation) or the confounding effects of socio-environmental and behavioral factors^4^.

Mendelian randomization (MR) is an approach that can help strengthen causal inference^5,6^ by using genetic variation associated with a risk factor of interest as an instrumental variable (IV). This approach attempts to reduce the limitations of observational studies by using genetic variants, which are randomly allocated at conception and not altered by disease^6,7^. Previous studies using MR have provided evidence supporting the causal role of alcohol consumption (as instrumented by the acetaldehyde dehydrogenase 2 [*ALDH2*] rs671 polymorphism; G > A) on CVD, predominantly in European populations. Whilst genetic variants within *ALDH2* have been used as IVs for alcohol intake in Asian populations^8^, only a few studies have done so in the context of CVD, due to the logistics and cost of collecting biological material, extracting DNA and performing genotyping in large enough populations^9–13^.

Alcohol flushing, known as the “Asian glow”, is associated with high levels of acetaldehyde and may indicate an individual’s sensitivity to alcohol^14^. This reaction is caused by an inherited deficiency in the ALDH2 enzyme, coded by the *ALDH2* gene, which acts to prevent the accumulation of toxic acetaldehyde during alcohol metabolism^15^. Alcohol flushing has been proposed to be a phenotypic marker of the *ALDH2* rs671 polymorphism in East Asian populations^14,16^. Further, the association of alcohol flushing and alcohol intake can be assumed to be independent of other confounders^17^. Therefore, it seems plausible to use alcohol flushing as an alternative phenotypic IV to evaluate the causal relationship between alcohol intake and CVD, whilst reducing the time and cost of genotyping required for classic MR methodology^18^. In fact, a study in 2003 used alcohol flushing as a phenotypic proxy for alcohol intake (instead of the *ALDH2* genotype) to determine the effect of alcohol on cancer^19^. In addition, one study used alcohol flushing as a phenotypic IV for alcohol intake in the context of CVD and hypertension^17^, but failed to compare results with those derived using a genetic variant (in this case, the *ALDH2* rs671 polymorphism) to validate alcohol flushing as an IV.

**Figure 1:**
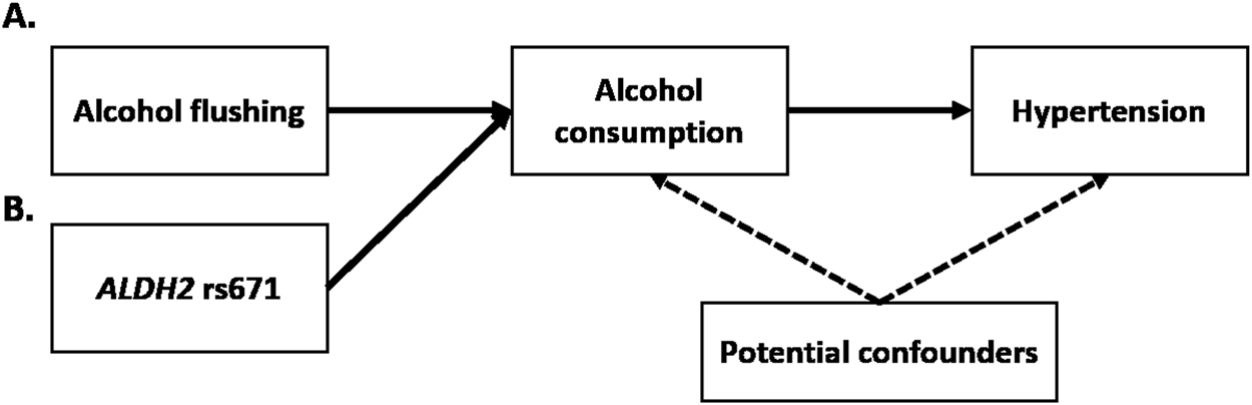
Directed acyclic graph showing the framework of this study. Alcohol flushing (**A**) and the *ALDH2* rs671 variant (**B**) were used as an instrumental variable for alcohol consumption to assess the causal role of alcohol consumption on hypertension risk.

We previously demonstrated an association between alcohol intake and an increased risk of hypertension in a Korean population using the *ALDH2* rs671 variant as an IV in a formal MR analyses^11^. To extend this finding, we aimed to test whether alcohol flushing could be used as an alternative phenotypic IV for alcohol intake (and be used as a valid proxy for the rs671 variant in the *ALDH2* gene) by comparing the IV results when using alcohol flushing with the MR results (i.e., using the rs671 genotype) within the same population, which estimated the effect of alcohol intake on BP and hypertension (Fig. 1).

## Results

### Baseline characteristics of study participants

Baseline characteristics of the participants are shown in Table 1. Monthly income level was associated with alcohol flushing in the total population and in men/women. Age was additionally associated with alcohol flushing in men (Table 1). There were no differences in characteristics of subjects according to the rs671 genotype, except for body mass index (BMI) in women only and the use of antihypertensive medications (Supplementary Table 1).

**Table 1:** Characteristics of study participants according to alcohol flushing status and gender.

Variables	Alcohol non-flushers (n = 1,330)	Alcohol flushers (n = 681)^1^	Beta coefficient; OR (95% CI)^2^	Men			Women		
				Alcohol non-flushers (n = 883)	Alcohol flushers (n = 470)	Beta coefficient; OR (95% CI)^2^	Alcohol non-flushers (n = 447)	Alcohol flushers (n = 211)	Beta coefficient; OR (95% CI) ^2^
Age (years)	55.5 ± 6.9	56.9 ± 7.4	1.383 (0.731, 2.035)	55.6 ± 6.7	57.5 ± 7.7	1.868 (1.074, 2.661)	55.4 ± 7.1	55.7 ± 6.6	0.287 (−0.855, 1.429)
Monthly household income (n, %)									
<1,000 USD	135 (10.2)	84 (12.3)	1.000 (ref)	71 (8.0)	53 (11.3)	1.000 (ref)	64 (14.3)	31 (14.7)	1.000 (ref)
1,000–2,000 USD	175 (13.2)	135 (19.8)	1.632 (1.275, 2.088)	101 (11.4)	86 (18.3)	1.734 (1.269, 2.370)	74 (16.6)	49 (23.2)	1.525 (1.017, 2.287)
2,000–4,000 USD	563 (42.3)	254 (37.3)	0.810 (0.670, 0.980)	370 (41.9)	189 (38.1)	0.853 (0.678, 1.073)	193 (43.2)	75 (35.6)	0.726 (0.517, 1.108)
≥6,000 USD	457 (34.4)	208 (30.5)	0.840 (0.689, 1.024)	341 (38.6)	152 (32.3)	0.760 (0.600, 0.962)	116 (26.0)	56 (26.5)	1.031 (0.711, 1.495)
Drinking									
Ex-drinker (n, %)	332 (25.0)	283 (51.6)	1.000 (ref)	137 (15.5)	166 (35.3)	1.000 (ref)	195 (43.6)	117 (55.5)	1.000 (ref)
Current drinker (n, %)	998 (75.0)	398 (58.4)	0.468 (0.384, 0.569)	746 (84.5)	304 (64.7)	0.336 (0.259, 0.437)	252 (56.4)	94 (44.6)	0.622 (0.447, 0.864)
Total alcohol intake (g/day)	16.2 ± 24.5	7.1 ± 16.9	−9.074 (−11.124, −7.023)	22.5 ± 27.3	9.6 ± 19.7	−12.831 (−15.625, −10.037)	3.9 ± 8.8	1.6 ± 3.5	−2.280 (−3.507, −1.054)
Smoking (n, %)									
Non-smoker	595 (44.7)	298 (43.8)	1.000 (ref)	166 (18.8)	93 (19.8)	1.000 (ref)	429 (96.0)	205 (97.2)	1.000 (ref)
Ex-smoker	457 (34.4)	258 (37.9)	1.165 (0.962, 1.411)	452 (51.2)	255 (54.3)	1.131 (0.904, 1.415)	5 (1.1)	3 (1.4)	1.275 (0.302, 5.386)
Current-smoker	278 (20.9)	125 (18.4)	0.851 (0.673, 1.076)	265 (30.0)	122 (26.0)	0.818 (0.636, 1.051)	13 (2.9)	3 (1.4)	0.482 (0.136, 1.708)
Physical activity	842 (63.3)	447 (65.6)	1.107 (0.912, 1.344)	554 (62.7)	313 (66.6)	1.184 (0.936, 1.498)	288 (64.4)	134 (63.5)	0.961 (0.683, 1.351)
MET-hours (hour/day)	42.2 ± 5.9	42.7 ± 7.4	0.489 (−0.110, 1.087)	42.4 ± 6.2	43.1 ± 8.2	0.729 (−0.050, 1.509)	41.7 ± 5.2	41.6 ± 5.4	−0.102 (−0.970, 0.766)
Adult height (cm)	163.5 ± 7.9	163.4 ± 8.2	−0.080 (−0.817, 0.656)	167.6 ± 5.5	167.4 ± 5.7	−0.245 (−0.866, 0.376)	155.4 ± 5.1	154.6 ± 5.5	−0.751 (−1.610, 0.107)
Medication use									
Anti-diabetic medications	136 (10.2)	75 (11.0)	1.087 (0.806, 1.464)	108 (12.2)	58 (12.3)	1.010 (0.719, 1.420)	28 (6.3)	17 (8.1)	1.311 (0.701, 2.453)
Anti-hypertensive medications	356 (26.8)	178 (26.1)	0.968 (0.785, 1.194)	247 (28.0)	125 (26.6)	0.933 (0.725, 1.200)	109 (24.4)	53 (25.1)	1.040 (0.712, 1.519)
Anti-dyslipidemic medications	70 (5.3)	43 (6.3)	1.213 (0.820, 1.795)	39 (4.4)	22 (6.7)	1.063 (0.622, 1.815)	31 (6.9)	21 (10.0)	1.483 (0.831, 2.649)
Genotype of rs671 in *ALDH2*									
GG/GA + AA (%)	92.0/8.0	41.3/58.7	—	91.5/8.5	37.0/63.0	—	93.1/6.9	50.7/49.3	—

OR, Odds ratio; CI, Confidence Interval; USD, US dollars; MET, metabolic equivalent.

^1^Values are means ± SD for continuous variables, or number (percentages) for categorical variables.

^2^Values were derived by logistic regression for the categorical variables (Odds ratio [95% Confidence Interval]) or by linear regression for the continuous variables (beta coefficient [95% Confidence Intervals]) and represent the change in each variable by alcohol flushing status.

### Association between instrumental variables and alcohol intake

Participants who were flushers consumed less alcohol than non-flushers (difference in alcohol intake with alcohol flushers vs. non-flushers: −9.07 g/day; 95% confidence interval [CI]: −11.12, −7.02; P-value: 8.3 × 10^−18^) (Table 1). Similarly, carriers of the rs671 A allele consumed less alcohol than non-carriers (difference in alcohol intake with the rs671 GA + AA vs. GG genotypes: −7.94 g/day; 95% CI: −10.20, −5.69; P-value: 6.1 × 10^−12^; Supplementary Table 1). Of the two IVs, there was little difference in terms of their strength and validity to predict alcohol intake (F-statistic for the rs671 genotype = 47.87 and alcohol flushing = 75.28, in the total population).

### Association between alcohol intake and hypertension

Alcohol intake was associated with an increased hypertension risk (odds ratio [OR] per 1 unit (g/day) increase in alcohol intake: 1.01; 95% CI: 1.00, 1.01; P-value: 2.0 × 10^−4^) and BP adjusted for treatment effect by adding 10 mmHg/15 mmHg for SBP and 5 mmHg/10 mmHg for DBP, respectively (difference per g/day increase in alcohol intake: 0.09 mmHg; 95% CI: 0.06, 0.12; P-value: 2.1 × 10^−7^ for systolic blood pressure [SBP] and difference: 0.06 mmHg; 95% CI: 0.04, 0.08; P-value: 7.2 × 10^−9^ for diastolic blood pressure [DBP]) in the total population (Table 2). In men, alcohol intake was associated with higher risk of hypertension and higher BP and, in women, there was marginal evidence for an effect of alcohol intake on hypertension and BP (Table 2).

**Table 2:** Association between alcohol intake (g/day) and hypertension.

**Disease**	Total (n = 2,011)		Men (n = 1,353)		Women (n = 658)	
	**OR (95% CI)** ^**1**^	**P-value** ^2^	**OR (95% CI)**	**P-value** ^2^	**OR (95% CI)**	**P-value** ^2^
Hypertension	1.008 (1.004, 1.013)	0.0002	1.007 (1.003, 1.012)	0.001	1.025 (1.002, 1.049)	0.032
**Blood pressure**	**Beta coefficient (95% CI)** ^**1**^	**P-value** ^2^	**Beta coefficient (95% CI)**	**P-value** ^2^	**Beta coefficient (95% CI)**	**P-value** ^2^
SBP (mmHg)	0.073 (0.043, 0.103)	1.4 × 10^−6^	0.068 (0.039, 0.097)	4.6 × 10^−6^	0.153 (−0.006, 0.313)	0.060
Adjusting treatment effect + 10mmHg^**3**^	0.085 (0.053, 0.117)	2.5 × 10^−7^	0.079 (0.047, 0.110)	8.8 × 10^−7^	0.178 (0.004, 0.352)	0.045
Adjusting treatment effect + 15mmHg^**3**^	0.090 (0.056, 0.124)	2.1 × 10^−7^	0.084 (0.051, 0.117)	8.2 × 10^−7^	0.191 (0.006, 0.376)	0.043
DBP (mmHg)	0.051 (0.033, 0.070)	7.9 × 10^−8^	0.046 (0.028, 0.065)	4.5 × 10^−6^	0.122 (0.028, 0.215)	0.011
Adjusting treatment effect + 5mmHg^**3**^	0.057 (0.037, 0.076)	1.1 × 10^−8^	0.052 (0.032, 0.071)	2.3 × 10^−7^	0.134 (0.035, 0.233)	0.008
Adjusting treatment effect + 10mmHg^**3**^	0.063 (0.042, 0.084)	7.2 × 10^−9^	0.057 (0.036, 0.078)	1.5 × 10^−7^	0.147 (0.037, 0.256)	0.009

OR, Odds ratio; CI, Confidence Interval; SBP, systolic blood pressure; DBP, diastolic blood pressure.

^**1**^Values are ORs (95% CI) for hypertension or beta coefficients (95% CI) for blood pressure per g/day increase in alcohol intake.

^2^P values were derived from regression analysis with adjustment for age, sex (for total subjects), income, MET-hour/day and smoking status. Non-normally distributed variables were log transformed for statistical analysis.

^3^To adjust treatment effect on blood pressure, sensible constants were added to the observed blood pressure values of all subjects on treatment (see Methods).

### Causal association between alcohol intake and hypertension

IV analyses provided some evidence that alcohol intake (as predicted by alcohol flushing) caused an increased hypertension risk (OR per g/day increase in alcohol intake: 1.02; 95% CI: 1.00, 1.05; P = 0.07) and BP adjusted for treatment effect (as above) in the total population (difference per g/day increase in alcohol intake: 0.17 mmHg; 95% CI: 0.0003, 0.35; P-value: 0.05 for SBP and difference: 0.14 mmHg; 95% CI: 0.03, 0.25; P-value: 0.01 for DBP) and with both an increased DBP (difference: 0.10 mmHg; 95% CI: 0.001, 0.19; P-value: 0.05) and increased prevalence of hypertension (OR: 1.02; 95% CI: 1.00, 1.04; P-value: 0.04) in men (Table 3). Whilst there was no strong evidence for an effect of alcohol intake (as predicted by alcohol flushing) on hypertension risk in women, the effect estimates of alcohol intake on BP were greater than those in men, but with wider confidence intervals.

**Table 3:** Instrumental variable estimates of alcohol intake (g/day) and hypertension based on alcohol flushing.

**Disease**	Total (n = 2,011)		Men (n = 1,353		Women (n = 658)	
	**OR (95% CI)** ^**1**^	**P-value** ^2^	**OR (95% CI)**	**P-value** ^2^	**OR (95% CI)**	**P-value** ^2^
Hypertension	1.022 (0.999, 1.045)	0.065	1.022 (1.001, 1.042)	0.037	0.983 (0.823, 1.175)	0.853
**Blood pressure**	**Beta coefficient (95% CI)** ^**1**^	**P-value**	**Beta coefficient (95% CI)**	**P-value**	**Beta coefficient (95% CI)**	**P-value**
SBP (mmHg)	0.123 (−0.027, 0.273)	0.107	0.067 (−0.062, 0.196)	0.309	0.626 (−0.564, 1.815)	0.303
Adjusting treatment effect + 10mmHg^**3**^	0.156 (−0.006, 0.318)	0.059	0.100 (−0.038, 0.239)	0.156	0.614 (−0.677, 1.904)	0.351
Adjusting treatment effect + 15mmHg^**3**^	0.173 (0.0004, 0.345)	0.050	0.117 (−0.030, 0.265)	0.119	0.608 (−0.756, 1.972)	0.383
DBP (mmHg)	0.109 (0.014, 0.203)	0.024	0.062 (−0.022, 0.146)	0.149	0.529 (−0.188, 1.246)	0.148
Adjusting treatment effect + 5mmHg^**3**^	0.125 (0.026, 0.224)	0.013	0.078 (−0.008, 0.165)	0.076	0.523 (−0.232, 1.277)	0.174
Adjusting treatment effect + 10mmHg^**3**^	0.142 (0.034, 0.250)	0.010	0.095 (0.001, 0.189)	0.048	0.517 (−0.303, 1.337)	0.217

OR, Odds ratio; CI, Confidence Interval; SBP, systolic blood pressure; DBP, diastolic blood pressure

^**1**^ORs and beta coefficients by instrumental variable (IV) estimation were obtained from IV regressions with a two-stage least squares estimation method (in logistic and linear regression models, respectively), using alcohol flushing as an instrument for alcohol intake. To predict the amount of alcohol intake, non-flushers were regarded as a reference group.

^2^P values were derived from IV regression analysis with adjustment for age, sex (for total subjects), income, MET-hour/day and smoking status.

^**3**^To adjust treatment effect on blood pressure, sensible constants were added to the observed blood pressure values of all subjects on treatment (see Methods).

MR results suggested that higher alcohol intake (as predicted by the rs671 GG genotype) caused a greater risk of hypertension (OR per g/day increase in alcohol intake: 1.04; 95% CI: 1.01, 1.06; P-value: 0.01) and higher BP adjusted for treatment effect (as above) (difference per g/day increase in alcohol intake: 0.32 mmHg; 95% CI: 0.12, 0.51; P-value: 0.001 for SBP and difference: 0.18 mmHg; 95% CI: 0.06, 0.30; P-value: 0.003 for DBP) in the total population (Table 4). In men, there was some evidence that higher alcohol intake (as instrumented by the rs671 genotype) caused higher risk of hypertension (OR: 1.02; 95% CI: 1.00, 1.04; P = 0.06) and higher levels of BP (difference: 0.18 mmHg; 95% CI: 0.01, 0.34; P-value: 0.03 for SBP and difference: 0.10 mmHg; 95% CI: −0.004, 0.20; P = 0.06 for DBP). In women, there was some evidence suggesting that higher alcohol intake caused a higher risk of hypertension (OR: 1.24; 95% CI: 0.97, 1.57; P = 0.09) and an elevated BP (difference: 2.09 mmHg; 95% CI: −0.09, 4.26; P = 0.06 for SBP and (difference: 1.13 mmHg; 95% CI: −0.10, 2.36; P = 0.07) to a greater magnitude than that observed in men, but with wider confidence intervals.

**Table 4:** Instrumental variable estimates of alcohol intake (g/day) and hypertension based on *ALDH2* rs671 genotype.

**Disease**	Total (n = 2,011)			Men (n = 1,353)			Women (n = 658)		
	**OR (95% CI)** ^**1**^	**P-value** ^2^	**P(het)** ^**3**^	**OR (95% CI)**	**P-value**	**P(het)** ^**3**^	**OR (95% CI)**	**P-value**	**P(het)** ^**3**^
Hypertension	1.035 (1.009, 1.061)	0.008	0.472	1.021 (0.999, 1.044)	0.058	0.993	1.235 (0.969, 1.574)	0.088	0.155
**Blood pressure** ^**4**^	**Beta coefficient (95% CI)** ^**1**^	**P-value**	**P(het)** ^**3**^	**Beta coefficient (95% CI)**	**P-value**	**P(het)** ^**3**^	**Beta coefficient (95% CI)**	**P-value**	**P(het)** ^**3**^
SBP (mmHg)	0.233 (0.068, 0.399)	0.006	0.332	0.118 (−0.023, 0.259)	0.101	0.601	1.713 (−0.134, 3.559)	0.069	0.332
Adjusting treatment effect + 10 mmHg	0.289 (0.108, 0.469)	0.002	0.284	0.157 (0.005, 0.309)	0.043	0.590	1.962 (−0.090, 4.013)	0.061	0.276
Adjusting treatment effect + 15 mmHg	0.316 (0.124, 0.509)	0.001	0.275	0.177 (0.014, 0.339)	0.033	0.596	2.086 (−0.091, 4.264)	0.060	0.259
DBP (mmHg)	0.124 (0.021, 0.226)	0.018	0.836	0.060 (−0.031, 0.151)	0.198	0.976	0.885 (−0.135, 1.906)	0.089	0.575
Adjusting treatment effect + 5 mmHg	0.151 (0.043, 0.259)	0.006	0.729	0.079 (−0.015, 0.174)	0.100	0.989	1.010 (−0.100, 2.120)	0.075	0.477
Adjusting treatment effect + 10 mmHg	0.179 (0.061, 0.297)	0.003	0.650	0.099 (−0.004, 0.202)	0.059	0.958	1.134 (−0.095, 2.364)	0.071	0.413

OR, Odds ratio; CI, Confidence Interval; SBP, systolic blood pressure; DBP, diastolic blood pressure

^**1**^ORs and beta coefficients by IV estimates were obtained by IV regression with a two-stage least squares estimation method (in logistic and linear regression models, respectively), using rs671 genotype as an instrument for alcohol intake (additive model; ref: AA).

^2^P values were derived from IV regression analysis with adjustment for age, sex (for total subjects), income, MET-hour/day and smoking status.

^**3**^Heterogeneity in estimates (p[het]) between instruments (alcohol flushing and genotype) was assessed by Cochran’s Q test with fixed effects.

^**4**^To adjust for treatment effect on blood pressure, sensitivity constants were added to the observed blood pressure values of all subjects on treatment (see Methods).

All results for BP were consistent and robust in a range of models constructed to control for the treatment effect on BP (by adding 10 mmHg/15 mmHg for SBP and 5 mmHg/10 mmHg for DBP, respectively).

The results from IV analyses using either alcohol flushing or the rs671 genotype as IVs were therefore consistent with regards to the causal effect of alcohol intake on BP and hypertension risk (Table 4), with MR analyses providing consistently greater effect estimates than those derived using alcohol flushing (P-value for heterogeneity between IV analyses >0.275 in the total population).

### Sensitivity analysis

Subjects carrying the A allele in rs671 had an increased prevalence of alcohol flushing in the dominant (OR: 17.35; 95% CI: 13.42, 22.44; P-value: <1.0 × 10^−18^), recessive (OR: 17.96; 95% CI: 4.12, 78.30; P-value: 1.0 × 10^−4^) and additive (OR: 16.10; 95% CI: 12.48, 20.77; P-value: <1.0 × 10^−18^) models (Supplementary Table 2). Sensitivity analyses including those who had reported being “never drinkers” but who had experienced flushing symptoms as “ever-drinkers” provided effect estimates that were slightly greater compared with those who were not considered “never-drinkers-but-flushers” (Supplementary Table 3), but, the direction of the estimates was unchanged in both men and women.

The genetic risk score (GRS) comprising both rs671 and rs1229984 SNPs was approximately normally distributed within this Korean population (See the Supplementary Method and Supplementary Fig. S1 for details), was not strongly associated with confounding factors and was associated with an increase in alcohol intake (Supplementary Table 4). Results from MR analysis using this GRS as an IV for alcohol intake were consistent with the main analyses, providing evidence that increased alcohol intake caused a higher risk of hypertension and higher levels of BP (Supplementary Table 5).

A comparison of alcohol intake between those who were homozygous for the G allele of the rs671 SNP with and without flushing indicated that the individuals who were GG homozygous and flushers drank less than those who were not flushers (Supplementary Table 6; −6.84 g/day; 95% CI: −9.90, −3.78; P-value: 1.0 × 10^−5^).

## Discussion

Within this study, we showed comparable causal effect estimates of alcohol intake on BP and hypertension risk in a sample of the Korean population, using both alcohol flushing and genetic variants associated with alcohol intake (including the *ALDH2* rs671 polymorphism [G > A]) as separate IVs. Of the IVs, there was little difference in terms of their strength and validity to predict alcohol intake. While confirming that alcohol intake was associated with an increased risk of hypertension, we demonstrated the possibility of using alcohol flushing as a marker of alcohol intake and as a valid proxy for the *ALDH2* genetic variant in the Korean population.

This study found that increased alcohol intake (as predicted by alcohol flushing and the *ALDH2* rs671 genotype) was likely to cause an increased BP in this Korean population sample. Using IV in this context allowed us to assess the causal relationship of alcohol intake with hypertension with more accurate estimates. Previous MR studies using the *ALDH2* rs671 polymorphism as an IV have reported similar results and thus validate the use of *ALDH2* rs671 as an IV in studies on the effects of alcohol intake on health-related traits^8,10,20,21^. In this investigation, which is an extension of the previous MR analysis with the rs671 genotype, we found that alcohol flushing was associated with modulation of alcohol intake in a Korean population, as alcohol flushing after drinking a small amount of alcohol is common in East Asians^22–24^. Observational studies in East Asian populations indicate that people with inactive ALDH2 enzyme (i.e., the presence of the rs671 A allele) have a high prevalence of facial flushing caused by the accumulation of acetaldehyde^25^. Individuals with the flushing response drink less alcohol and also have a lower risk of hypertension as compared to those without the flushing response^15^.

Further, we demonstrated that alcohol flushing may be a reliable IV for alcohol intake, as well as a proxy for the *ALDH2* rs671 genotype. Our results from regression analyses suggest that the *ALDH2* rs671 SNP and flushing were strongly associated (Supplementary Table 2), supporting previous evidence that alcohol flushing had a sensitivity and specificity of approximately 90% for predicting *ALDH2* genetic variation in East Asian population^19,26^. We found that alcohol flushing satisfied three core assumptions for an IV. In our data, alcohol flushing was not associated with a majority of confounding factors including sex, smoking, physical activity and medication use. To control for the potentially confounding effect of age and income (which were associated with alcohol flushing), these variables were included in statistical models. Additionally, we found that the prevalence of alcohol flushing was related to a decreased alcohol intake. Finally, alcohol flushing is most likely to influence BP only through alcohol intake, since the alcohol flushing symptom appears after alcohol intake.

MR analyses provide causal estimates of life-long exposure to alcohol use, by comparing individuals according to their genotype, which are randomly allocated at conception^18^. In comparison, alcohol flushing is an adverse side-effect of alcohol intake, which only occurs after an individual has started drinking (i.e., during adulthood). Therefore, the use of alcohol flushing as an instrument may only reflect an effect of alcohol intake from a specific period of the life course (i.e., in adulthood)^27,28^. Additionally, genotyping is likely to be more accurate than a questionnaire for flushing screening. The magnitude of the associations was expectedly greater in the MR analysis compared to the IV analysis using alcohol flushing as an IV. Despite greater effect sizes, the lack of statistical evidence for an effect of alcohol intake on adverse cardiovascular health in women is consistent with earlier finding^17^, but might be attributable to small sample size of women (since women drink far less than men), as reflected in the wider confidence intervals.

This finding highlights the possibility of using alcohol flushing as an IV to examine causality between alcohol intake and health-related traits in this Korean population, as the *ALDH2* rs671 genotype and alcohol flushing were of comparable strength. Furthermore, using alcohol flushing as an IV has important implications and should be highlighted. Most importantly, a simple questionnaire about alcohol flushing as a proxy of the ALDH2 enzyme function is reliable in detecting ALDH2 deficiency with high sensitivity^19,29,30^. This simple screening test for inactive ALDH2 based on alcohol flushing allows identification of ALDH2 function at a population-level in a simple, cost-effective and non-invasive manner^31^ and makes it possible to begin large-scale studies on of alcohol-related health problems without collecting biological specimens required for MR methodology.

The study also has some limitations. First, the *ALDH2* rs671 polymorphism was imputed rather than genotyped directly. However, post-imputation quality control indicated that the rs671 genotype in *ALDH2* was good quality (imputation information value ≥ 0.9). Secondly, although the questionnaires for alcohol flushing status have been validated, misclassification is possible. Some individuals who reported being “never-drinkers” also reported that they had experienced flushing symptoms. To control for this, we performed a sensitivity analysis that included “never-drinkers-but-flushers” in an “ever-drinker” group. Although this limitation would serve to attenuate the magnitude of our effect measures toward null, it is reassuring that the results of the sensitivity analyses were consistent with those in the main analyses. Thirdly, there is trend for a lowering BP in the patients who receive drug treatment, which attenuates the association between exposure and outcome. We controlled for treatment effect among subjects who took anti-hypertensive agents to prevent underestimation of the effect of alcohol intake on BP, as previously described^32^. After adjusting for such treatment effects, results were also consistent with those in the main analyses. Fourthly, although some alcohol flushers did not carry the A allele of the rs671 variant in the *ALDH2* gene, flushing was associated with decreased alcohol consumption (Supplementary Table 6 and Supplementary Table 7). This suggests that alcohol flushing can be used as an IV, as the associations of both instruments (flushing and the rs671 variant) with alcohol intake were comparably strong enough for the IV estimates to be unbiased^33^. Finally, there may be a lack of power to detect causal associations in this population (especially in sex-specific analyses) due to the relatively small sample size. Although some results had wide confidence intervals, effect estimates were consistent between methodologies used and therefore provide some evidence for a causal relationship^34,35^. Further work with larger samples is needed to confirm these associations and the validity of alcohol flushing as an IV for alcohol intake.

In conclusion, we replicated the causal effect of alcohol intake and hypertensive risk in a Korean population, which adds to our understanding of risk factors for CVD and related traits. This study also demonstrated the utility of alcohol flushing as a valid IV in comparison to using genetic variation at the *ALDH2* gene (rs671 polymorphism), which may therefore be a convenient and cost-effective alternative to investigating the causal effects of alcohol intake on health outcomes.

## Methods

### Study subjects

The subjects were enrolled from the Ansan-Ansung study, a part of the Korean Genome and Epidemiology Study (KoGES). Detailed information on the KoGES Ansan-Ansung study population is available elsewhere^36^. Briefly, the Ansan-Ansung study is a biannual survey with the baseline recruitment in 2001–2002 that continued through a sixth follow-up in 2013–2014. It is an ongoing evaluation of the effects of genetic and environmental risk factors on disease outcomes in the Korean population. The participants were 40–69 years of age at baseline recruitment and were residents of urban (Ansan) and rural (Ansung) areas selected from the national health examinee registry. This study evaluated data from the fourth (2009–2010) follow-up of participants only from the urban region (Ansan) because of the availability of data on alcohol flushing. Out of the 3,031 participants from Ansan, those without data on exposures and outcomes (e.g., alcohol flushing, alcohol intake, rs671 genotype and hypertension) and possible confounders (e.g., smoking, exercise and income) were excluded. People who had never been alcohol drinkers were also excluded because alcohol flushing only appears in those who have a history of alcohol intake. Ex-drinkers and current drinkers were included in the analyses. People who developed any type of cancer during the study period were also excluded. After the exclusions, the remaining 2,011 people were included in the study. All participants signed an informed consent form that was approved by the Human Subjects Review Committee at the Korea University Ansan Hospital or the Ajou University Medical center. The current study was approved by the Institute Review Board of the Korea University (No. KU-IRB-15-EX-256-A-1). The dataset analysed during the current study are not publicly available to preserve the privacy of participants but are available from the corresponding author upon request.

### Basic characteristics

Demographic characteristics of the study participants including age, income, physical activity and smoking status were collected during health interviews. Participants’ monthly income was stratified by United States (US) currency as <1,000 US Dollor (USD; 1,000,000 Korean Won) 1,000–2,000 USD, 2,000–4,000 USD and ≥6,000 USD. Participants who answered “Yes” to the question “Do you exercise at least once a week?” were regarded as regular exercisers and were then asked to report the time spent daily in sedentary, very light, light, moderate and vigorous activity. Participants were asked for details on each category of activity. Specific metabolic equivalent (MET) values were calculated by multiplying the hours reported spent in each category to yield a MET-hours score (0 for sleep or sedentary, 1.5 for very light, 2.4 for light, 5.0 for moderate and 7.5 for vigorous activity). Participants were considered to be current smokers if they had smoked cigarettes during the survey period.

### Alcohol traits

People who responded “No” to a question asking if they currently do not drink alcohol were considered to be current alcohol drinkers. Ex-drinkers were defined as subjects who had quit drinking before the baseline survey. Participants who indicated on the baseline, second or third follow-up questionnaires that they had consumed alcohol at least once were considered ex-drinkers. Total alcohol intake (g/day) was calculated using information on the average alcohol content of each type of alcoholic beverage. As the alcohol intake (g/day) was evaluated only in current alcohol consumers, the amount of alcohol consumed by ex-drinkers was recorded as zero. To investigate the occurrence of alcohol flushing, participants were asked the question “Have you ever experienced facial flushing after drinking a little alcohol – about a single cup (200 ml) of beer?” All questionnaires were provided in the Korean language.

### Blood pressure and disease outcome

BP was measured with a mercury sphygmomanometer after the participants had rested for at least 5 minutes in a sitting position. Four measurements were obtained, recorded to the nearest 2 mmHg and averaged. Participants with self-reported diagnosed hypertension, use of BP-lowering medication or those with an average measured systolic blood pressure (SBP) >140 mmHg or diastolic blood pressure (DBP) >90 mmHg were considered to be hypertensive.

### Genotyping of the rs671 variant

Peripheral blood samples were collected from the study participants at baseline and then genotyped using an Affymetrix Genome-Wide Human SNP array 5.0. Detailed information is provided elsewhere^37^. Missing genotypes were imputed statistically using the IMPUTE software based on the 1000 Genomes project^38^. As a post-imputation quality control, single-nucleotide polymorphisms (SNPs) were removed if the minor allele frequency was low (<0.05), the SNP call rate was low (<0.95), Hardy–Weinberg equilibrium was not satisfied (P < 1 × 10^−6^) or INFO was less than 0.9.

### Statistical analysis

Descriptive statistics of all the variables were reported as mean ± standard deviation (SD) for continuous variables and as number of counts and percentages for categorical variables in men and women, separately, by alcohol flushing status (Table 1) and rs671 genotype (Supplementary Table 1). The differences between participants carrying major homozygotes for rs671 (GG) and those who carried the minor allele (GA + AA) was determined by the *t*-test and the chi-square test. The associations of baseline characteristics and alcohol flushing were evaluated separately in men and women by regression models that included demographic factors and alcohol traits as the dependent variables and alcohol flushing as the independent variable. Results were presented as beta (β)-coefficients with 95% confidence intervals (CIs).

The association between alcohol flushing and BP was determined using an ordinary least square (OLS) regression model adjusting for the potential confounders (sex, age, income, MET and smoking status). The associations of alcohol flushing and hypertensive outcomes were tested separately in men and women. Results were reported as β-coefficients with 95% CIs for BP and odds ratios (ORs) with 95% CIs for hypertension risk. To adjust for the treatment effect on BP, we used a fixed addition method as previously described by Cui *et al*.^32^, adding average treatment effects (10 mmHg/15 mmHg for SBP and 5 mmHg/10 mmHg for DBP) to the treated pressure of men and women, respectively.

The causal effect of alcohol intake on BP and hypertension risk was evaluated using the IV analysis with a two-stage least squares estimation method separately in men and women, using alcohol flushing and the rs671 genotype as the phenotypic and genotypic instruments, respectively. For hypertension, a two-stage logistic model was used. In the first stage, alcohol intake was predicted by alcohol flushing or the rs671 genotype (GG vs. GA + AA) with adjustment for potential variables (sex, age, income, MET and smoking status) using a linear regression model. In the second stage, hypertension risk was predicted by fitting the alcohol intake value from the first stage, under a logistic regression model with adjustment for the same confounders as in the first stage. For BP, a two-stage linear model was applied using ivregress in Stata, similarly adjusting for confounders. The values were reported as ORs with 95% CIs for hypertension and β-coefficients with 95% CIs for BP. The differences of estimates between two instruments (p[het]) was evaluated by Cochran’s Q test using fixed effect model. We examined the strength and validity of each instrument using the F-statistic of the association of each instrument on alcohol intake (with an F-statistic >10 indicating adequate strength).

The sensitivity analyses were performed to control for any potential misclassification of alcohol flushing, since alcohol flushing status was determined by self-reporting questionnaires. Since some individuals who reported being “never-drinkers” but also reported experiences of flushing symptoms, we screened the Ansan-Ansung cohort data for these “never-drinkers-but-flushers” and performed the same IV analysis, including “never-drinkers-but-flushers” in an “ever-drinker” group. Additionally, we compared alcohol intake between individuals who were homozygous for the G allele of the rs671 SNP with and without flushing to demonstrate whether alcohol flushing can predict alcohol consumption in absence of the *ALDH2* rs671 variant.

The distribution of variable values and outliers were investigated by visual inspection. Statistical analyses were performed using Stata 14.0 (Stata Corp, College Station, TX, USA).

## Electronic supplementary material

Supplementary information

## References

[1] Global Burden of Disease Risk Factors Collaborators (2015). Global, regional, and national comparative risk assessment of 79 behavioural, environmental and occupational, and metabolic risks or clusters of risks in 188 countries, 1990–2013: a systematic analysis for the Global Burden of Disease Study 2013. Lancet.

[2] Chagas P (2016). Association of alcohol consumption with coronary artery disease severity. Clin. Nutr..

[3] Ikehara S (2008). Alcohol Consumption and Mortality From Stroke and Coronary Heart Disease Among Japanese Men and Women The Japan Collaborative Cohort Study. Stroke.

[4] Emberson JR, Bennett DA (2006). Effect of alcohol on risk of coronary heart disease and stroke: causality, bias, or a bit of both?. Vasc Health Risk Manag.

[5] Davey Smith G, Hemani G (2014). Mendelian randomization: genetic anchors for causal inference in epidemiological studies. Hum. Mol. Genet..

[6] Davey Smith G, Ebrahim S (2003). ‘Mendelian randomization’: can genetic epidemiology contribute to understanding environmental determinants of disease?. Int. J. Epidemiol..

[7] Sheehan NA, Didelez V, Burton PR, Tobin MD (2008). Mendelian randomisation and causal inference in observational epidemiology. PLoS Med..

[8] Yeung SLA (2013). Is aldehyde dehydrogenase 2 a credible genetic instrument for alcohol use in Mendelian randomization analysis in Southern Chinese men?. Int. J. Epidemiol..

[9] Yeung SLA (2015). Evaluation of Moderate Alcohol Use With QT Interval and Heart Rate Using Mendelian Randomization Analysis Among Older Southern Chinese Men in the Guangzhou Biobank Cohort Study. Am. J. Epidemiol..

[10] Yeung SLA (2013). Moderate Alcohol Use and Cardiovascular Disease from Mendelian Randomization. PLoS One.

[11] Cho Y (2015). Alcohol intake and cardiovascular risk factors: A Mendelian randomisation study. Sci. Rep..

[12] Tabara Y (2016). The causal effects of alcohol on lipoprotein subfraction and triglyceride levels using a Mendelian randomization analysis: The Nagahama study. Atherosclerosis.

[13] Tabara Y (2016). Mendelian randomization analysis in three Japanese populations supports a causal role of alcohol consumption in lowering low-density lipid cholesterol levels and particle numbers. Atherosclerosis.

[14] Jung JG, Kim JS, Oh MK (2010). The Role of the Flushing Response in the Relationship Between Alcohol Consumption and Insulin Resistance. Alcoholism-Clinical and Experimental Research.

[15] Crabb DW, Edenberg HJ, Bosron WF, Li TK (1989). Genotypes for aldehyde dehydrogenase deficiency and alcohol sensitivity. The inactive ALDH2(2) allele is dominant. J. Clin. Invest..

[16] Harada S, Agarwal DP, Goedde HW (1981). Aldehyde Dehydrogenase-Deficiency as Cause of Facial Flushing Reaction to Alcohol in Japanese. Lancet.

[17] Yun KE (2017). Alcohol and coronary artery calcification: an investigation using alcohol flushing as an instrumental variable. Int. J. Epidemiol..

[18] Zuccolo L, Holmes MV (2017). Commentary: Mendelian randomization-inspired causal inference in the absence of genetic data. Int. J. Epidemiol..

[19] Yokoyama T (2003). Alcohol flushing, alcohol and aldehyde dehydrogenase genotypes, and risk for esophageal squamous cell carcinoma in Japanese men. Cancer Epidemiol. Biomarkers Prev..

[20] Taylor AE (2015). Exploring causal associations of alcohol with cardiovascular and metabolic risk factors in a Chinese population using Mendelian randomization analysis. Sci. Rep..

[21] Chen L, Davey Smith G, Harbord RM, Lewis SJ (2008). Alcohol intake and blood pressure: a systematic review implementing a Mendelian randomization approach. PLoS Med..

[22] Wall TL, Thomasson HR, Schuckit MA, Ehlers CL (1992). Subjective feelings of alcohol intoxication in Asians with genetic variations of ALDH2 alleles. Alcohol. Clin. Exp. Res..

[23] Quertemont E (2005). The role of acetaldehyde in the central effects of ethanol. Alcohol. Clin. Exp. Res..

[24] Brooks PJ, Enoch MA, Goldman D, Li TK, Yokoyama A (2009). The alcohol flushing response: an unrecognized risk factor for esophageal cancer from alcohol consumption. PLoS Med..

[25] Peng GS, Chen YC, Wang MF, Lai CL, Yin SJ (2014). ALDH2*2 but not ADH1B*2 is a causative variant gene allele for Asian alcohol flushing after a low-dose challenge: correlation of the pharmacokinetic and pharmacodynamic findings. Pharmacogenet. Genomics.

[26] Yokoyama A (2006). Esophageal squamous cell carcinoma and aldehyde dehydrogenase-2 genotypes in Japanese females. Alcohol. Clin. Exp. Res..

[27] Knott CS, Coombs N, Stamatakis E, Biddulph JP (2015). All cause mortality and the case for age specific alcohol consumption guidelines: pooled analyses of up to 10 population based cohorts. BMJ.

[28] Ng Fat L, Cable N, Shelton N (2015). Worsening of health and a cessation or reduction in alcohol consumption to special occasion drinking across three decades of the life course. Alcohol. Clin. Exp. Res..

[29] Asakage T (2007). Genetic polymorphisms of alcohol and aldehyde dehydrogenases, and drinking, smoking and diet in Japanese men with oral and pharyngeal squamous cell carcinoma. Carcinogenesis.

[30] Yokoyama A (1997). Reliability of a flushing questionnaire and the ethanol patch test in screening for inactive aldehyde dehydrogenase-2 and alcohol-related cancer risk. Cancer Epidemiol. Biomarkers Prev..

[31] Yokoyama T (2008). Health risk appraisal models for mass screening of esophageal cancer in Japanese men. Cancer Epidemiol. Biomarkers Prev..

[32] Cui JS, Hopper JL, Harrap SB (2003). Antihypertensive treatments obscure familial contributions to blood pressure variation. Hypertension.

[33] Stock JH, Wright JH, Yogo M (2002). A survey of weak instruments and weak identification in generalized method of moments. J Bus Econ Stat.

[34] Wasserstein RL, Lazar NA (2016). The ASA’s Statement on p-Values: Context, Process, and Purpose. Am Stat.

[35] Sterne JA, Davey Smith G (2001). Sifting the evidence-what’s wrong with significance tests?. BMJ.

[36] Kim Y, Han BG, KoGES group (2016). Cohort Profile: The Korean Genome and Epidemiology Study (KoGES) Consortium. Int. J. Epidemiol..

[37] Cho YS (2009). A large-scale genome-wide association study of Asian populations uncovers genetic factors influencing eight quantitative traits. Nat. Genet..

[38] 1000 Genomes Project Consortium (2015). A global reference for human genetic variation. Nature.

