# Supplementary material for: Exploring the utility of alcohol flushing as an instrumental variable for alcohol intake in Koreans
Source: Sci Rep. 2018 Jan 11;8:458. doi: 10.1038/s41598-017-18856-z (PMC5765011; doi:10.1038/s41598-017-18856-z)
Supplement: Supplementary file 1 — Supplementary information [file 41598_2017_18856_MOESM1_ESM.pdf]

Supplementary Information file

Exploring the utility of alcohol flushing as an instrumental variable for alcohol intake in Koreans

Yoonsu Cho, PhD [1,2]; Soyoung Kwak, Ms [1]; Sarah J. Lewis, PhD [2]; Kaitlin H. Wade, PhD [2]; Caroline L. Relton, PhD [2], George Davey Smith, MD [2]*, Min-Jeong Shin, PhD [1]*

[1] Department of Public Health Sciences, BK21PLUS Program in Embodiment: Health-Society Interaction, Graduate School, Korea University, Seoul, Republic of Korea. [2] MRC Integrative Epidemiology Unit, Population Health Sciences, Bristol Medical School, University of Bristol, Bristol, UK.

Supplementary Method

Construction of the Genetic Risk Score and Mendelian randomization analysis

We constructed a genetic risk score (GRS) for each individual based on the number of risk alleles of both *ALDH2* rs671 and *ADH1B* rs1229984, previously related to alcohol flushing, which allowed us to achieve higher power in Mendelian randomization (MR) analyses. The extracted SNP data are described in Supplementary Table 4. We used the generated GRS as the instrumental variable in MR analyses to assess the causal role of alcohol intake on the hypertensive outcomes, using the same statistical methods as for main analyses.

Supplementary Figure legend

Supplementary Figure 1. Distribution of genetic risk score in the study sample. The frequencies of genetic risk score (GRS) were approximately within normal distribution. The means of GRS were 2.17 ± 0.77 in the alcohol flusher group and 1.56 ± 0.67 in the alcohol non-flusher group, respectively.

.

Supplementary Figure 1.

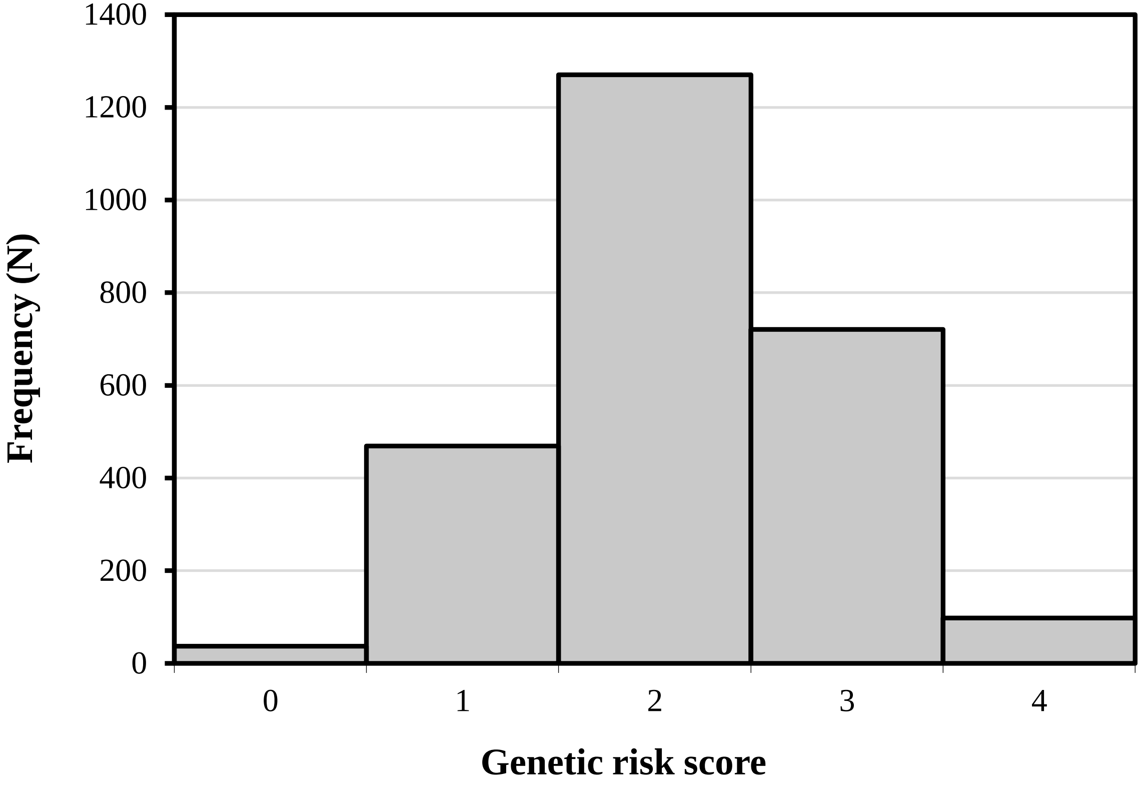

Supplementary Table 1. Characteristics of study participants according to rs671 genotype and gender.

Variables	Total[1]			Men[1]			Women[1]		
	GG (n=1,505)	GA+AA (n=506)[1]	Beta coefficient; OR (95% CI)[2]	GG (n=982)	GA+AA (n=371)	Beta coefficient; OR (95% CI)[2]	GG (n=523)	GA+AA (n=135)	Beta coefficient; OR (95% CI)[2]
Age (years)	56.0 ± 7.0	56.1 ± 7.3	0.078 (-0.636, 0.792)	56.1 ± 7.0	56.6 ± 7.5	0.509 (-0.344, 1.362)	55.7 ± 7.1	54.5 ± 6.3	-1.212 (-2.529, 0.104)
BMI (kg/m^2)	24.7 ± 2.8	24.5 ± 2.7	-0.232 (-0.511, 0.047)	24.7 ± 2.7	24.6 ± 2.7	-0.093 (-0.414, 0.228)	24.7 ± 3.0	24.1 ± 2.6	-0.610 (-1.168, -0.052)
Monthly household income (n, %)									
<1,000 USD	172 (11.4)	47 (9.3)	1.000 (ref)	91 (9.3)	33 (8.9)	1.000 (ref)	81 (15.5)	14 (10.4)	1.000 (ref)
1,000-2,000 USD	217 (14.4)	93 (18.4)	1.337 (1.023, 1.746)	126 (12.8)	61 (16.4)	1.337 (0.959, 1.864)	91 (17.4)	32 (23.7)	1.475 (0.934, 2.328)
2,000–4,000 USD	615 (40.9)	202 (39.9)	0.962 (0.783, 1.181)	397 (40.4)	152 (41.0)	1.023 (0.802, 1.304)	218 (41.7)	50 (37.0)	0.823 (0.557, 1.216)
≥6,000 USD	514 (33.3)	164 (32.4)	0.961 (0.775, 1.191)	368 (37.5)	125 (33.7)	0.848 (0.660, 1.090)	133 (25.4)	39 (28.9)	1.191 (0.782, 1.815)
Drinking									
Ex-drinker (n, %)	394 (26.2)	221 (43.7)	1.000 (ref)	160 (16.3)	143 (38.5)	1.000 (ref)	234 (44.7)	78 (57.8)	1.000 (ref)
Current drinker (n, %)	1,111 (73.8)	285 (56.3)	0.457 (0.371, 0.564)	822 (83.7)	228 (61.5)	0.310 (0.237, 0.406)	289 (55.3)	57 (42.2)	0.592 (0.404, 0.867)
Total alcohol intake (g/day)	15.1 ± 23.8	7.2 ± 17.5	-7.943 (-10.195, -5.692)	21.3 ± 26.8	9.2 ± 19.9	-12.098 (-15.100, -9.096)	3.5 ± 8.2	1.6 ± 4.1	-1.911 (-3.336, -0.486)
Alcohol flushing (n, %)	281 (18.7)	400 (79.1)	-	174 (17.7)	296 (79.8)	-	107 (20.5)	104 (77.0)	-
Smoking (n, %)									
Non-smoker	684 (45.5)	209 (41.3)	1.000 (ref)	183 (18.6)	76 (20.5)	1.000 (ref)	501 (95.8)	133 (98.5)	1.000 (ref)
Ex-smoker	520 (34.6)	195 (27.3)	1.188 (0.964, 1.463)	513 (52.2)	194 (52.3)	1.002 (0.789, 1.273)	7 (1.3)	1 (0.7)	0.550 (0.067, 4.510)
Current-smoker	301 (20.0)	102 (20.2)	1.010 (0.786, 1.298)	286 (29.1)	101 (27.2)	0.910 (0.697, 1.189)	15 (2.9)	1 (0.7)	0.253 (0.033, 1.930)
Physical activity	950 (63.1)	339 (67.0)	1.186 (0.959, 1.467)	617 (62.8)	250 (67.4)	1.222 (0.949, 1.574)	333 (63.7)	89 (65.9)	1.104 (0.741, 1.644)
MET-hours (hour/day)	42.3 ± 6.3	42.5 ± 7.0	0.265 (-0.388, 0.917)	42.6 ± 6.7	42.9 ± 7.5	0.316 (-0.516, 1.149)	41.7 ± 5.3	41.6 ± 5.3	-0.139 (-1.142, 0.863)
Adult height (cm)	163.3 ± 8.1	164.0 ± 7.6	0.742 (-0.060, 1.544)	167.6 ± 5.5	167.2 ± 5.6	-0.483 (-1.145, 0.180)	155.1 ± 5.2	155.4 ± 5.4	0.300 (-0.694, 1.294)
Medication use									
Anti-diabetic medications	168 (11.2)	43 (8.5)	0.739 (0.520, 1.050)	127 (12.9)	39 (10.5)	0.791 (0.541, 1.157)	41 (7.8)	4 (3.0)	0.359 (0.126, 1.020)
Anti-hypertensive medications	418 (27.8)	116 (22.9)	0.773 (0.611, 0.979)	281 (28.6)	91 (24.5)	0.811 (0.616, 1.066)	137 (26.2)	25 (18.5)	0.640 (0.398, 1.031)
Anti-dyslipidemic medications	82 (5.5)	31 (6.1)	1.133 (0.740, 1.734)	42 (4.3)	19 (5.1)	1.208 (0.693, 2.106)	40 (7.7)	12 (8.9)	1.178 (0.600, 2.313)

OR, Odds ratio; CI, confidence interval; USD, US dollars; MET, metabolic equivalent.

[1] Values are means ± SD for continuous variables, or number (percentages) for categorical variables.

[2] Values were derived by logistic regression for the categorical variables (Odds ratio [95% Confidence Intervals]) or by linear regression for the continuous variables (beta coefficient [95% Confidence Intervals]) and represent the change in each variable by the rs671 genotype status (GA+AA vs. GG).

Supplementary Table 2. Association of rs671 genotype with flushing among Korean men and women.

**Ever drinkers**	**Total (n=2,011)**			**Men (n=1,112)**			**Women (n=535)**		
	**N**	**OR (95% CI)**	**P-value**	**N**	**OR (95% CI)**	**P-value**	**N**	**OR (95% CI)**	**P-value**
***ALDH2* rs671**									
GG	1,505	1.00 (ref)		982	1.00 (ref)		523	1.00 (ref)	
GA	487	16.9 (13.04, 21.90)	$<1.0\times10^{-18}$	356	18.70 (13.68, 25.56)	$<1.0\times10^{-18}$	131	13.23 (8.29, 21.11)	$<1.0\times10^{-18}$
AA	19	41.28 (9.42, 180.98)	8.1×10^{-7}	15	73.39 (9.51, 566.57)	3.8×10^{-5}	4	13.63 (1.38, 134.98)	0.026
Dominant model (GG vs GA+AA)	2,011	17.35 (13.42, 22.44)	$<1.0\times10^{-18}$	1,112	19.41 (14.23, 26.48)	$<1.0\times10^{-18}$	535	13.24 (8.35, 21.01)	$<1.0\times10^{-18}$
Recessive model (GG+GA vs AA)	2,011	17.96 (4.12, 78.30)	0.0001	1,112	29.40 (3.83, 225.53)	0.001	535	7.69 (0.78, 75.43)	0.080
Additive model	2,011	16.10 (12.48, 20.77)	$<1.0\times10^{-18}$	1,112	18.10 (13.30, 24.64)	$<1.0\times10^{-18}$	535	12.13 (7.69, 19.12)	$<1.0\times10^{-18}$

OR, Odds ratio; CI, confidence interval

[1]ORs were obtained by logistic regression using rs671 genotype as an exposure. The reference genotype in the additive model was GG. All regression models were adjusted for age, sex (for total subjects), income, MET-hour/day and smoking status and represent the odds of being an alcohol flusher (vs. non-flusher) with each genotype and model of the rs671 genetic variant.

Supplementary Table 3. Instrumental variable estimates of alcohol intake (g/day) and hypertension based on alcohol flushing including those categorized as "never-drinkers-but-flushers".

	Total (n=2,595)		Men (n=1,468)		Women (n=1,127)	
**Diseases**	**OR (95% CI)[1]**	**P-value[2]**	**OR (95% CI)**	**P-value[2]**	**OR (95% CI)**	**P-value[2]**
Hypertension	1.023 (1.001, 1.045)	0.040	1.022 (1.005, 1.040)	0.012	0.994 (0.841, 1.174)	0.941
**Blood pressure**	**Beta coefficient (95% CI)**	**P-value**	**Beta coefficient (95% CI)**	**P-value**	**Beta coefficient (95% CI)**	**P-value**
SBP (mmHg)	0.117 (-0.022, 0.256)	0.099	0.052 (-0.059, 0.164)	0.359	0.694 (-0.372, 1.760)	0.202
Adjusting treatment effect +10mmHg[3]	0.151 (0.001, 0.302)	0.048	0.088 (-0.032, 0.208)	0.149	0.690 (-0.462, 1.842)	0.241
Adjusting treatment effect +15mmHg[3]	0.169 (0.009, 0.328)	0.039	0.106 (-0.021, 0.234)	0.102	0.688 (-0.530, 1.906)	0.268
DBP (mmHg)	0.116 (0.028, 0.204)	0.010	0.073 (0.001, 0.146)	0.046	0.459 (-0.181, 1.098)	0.160
Adjusting treatment effect +5mmHg[3]	0.133 (0.041, 0.225)	0.005	0.091 (0.016, 0.167)	0.017	0.457 (-0.216, 1.129)	0.183
Adjusting treatment effect +10mmHg[3]	0.151 (0.050, 0.251)	0.003	0.110 (0.028, 0.191)	0.009	0.455 (-0.278, 1.188)	0.224

OR, odds ratio; CI, confidence interval; SBP, systolic blood pressure; DBP, diastolic blood pressure

[1] ORs and beta coefficients by instrumental variable (IV) estimation were obtained from IV regressions with a two-stage least squares estimation method (in logistic and linear regression models, respectively), using alcohol flushing as an instrument for alcohol intake. To predict the amount of alcohol intake, non-flushers were regarded as a reference group.

[2] P values were derived from IV regression analysis with adjustment for age, sex (for total subjects), income, MET-hour/day and smoking status.

[3] To adjust treatment effect on blood pressure, sensible constants were added to the observed blood pressure values of all subjects on treatment (see Methods).

Supplementary Table 4. Association of genetic risk score with characteristics of study participants according to gender.

	Total (n=2,011)		Men (n=1,353)		Women (n=658)	
**Genotypes**	**Percentage or mean [2]**		**Percentage or mean**		**Percentage or mean**	
rs671 in *ALDH2* (GG / AG+AA, %)	74.8 / 25.2		72.6 / 27.4		79.5 / 20.5	
rs1229984 in *ADH1B* (TT+TC / CC, %)	94.0 / 6.0		93.9 / 6.1		94.2 / 5.8	
Genetic risk score (mean ± SD)[1]	2.23 ± 0.77		2.19 ± 0.77		2.31 ± 0.74	
**Variables**	**OR / Beta coefficient (95% CI) [3]**	**P-value**	**OR / Beta coefficient (95% CI)**	**P-value**	**OR / Beta coefficient (95% CI)**	**P-value**
Age (years)	0.066 (-0.339, 0.471)	0.751	0.002 (-0.490, 0.494)	0.994	0.315 (-0.405, 1.034)	0.390
Monthly household income (n, %)						
<1,000 USD	1.000 (ref)	-				
1,000-2,000 USD	0.920 (0.785, 1.078)	0.301	0.928 (0.760, 1.133)	0.463	0.862 (0.661, 1.124)	0.272
2,000–4,000 USD	0.999 (0.889, 1.122)	0.985	1.014 (0.881, 1.166)	0.848	0.965 (0.782, 1.191)	0.741
≥6,000 USD	1.031 (0.913, 1.164)	0.625	1.014 (0.879, 1.169)	0.853	1.166 (0.921, 1.476)	0.201
Drinking						
Ex-drinker	1.000 (ref)	-				
Current-drinker	1.256 (1.108, 1.424)	0.0004	1.585 (1.333, 1.884)	1.8 x $\times 10^{-7}$	1.078 (0.876, 1.325)	0.478
Total alcohol intake (g/day)	2.828 (1.542, 4.114)	0.00002	4.690 (2.939, 6.441)	1.7 x $\times 10^{-7}$	0.813 (0.034, 1.591)	0.041
Smoking (n, %)						
Non-smoker	1.000 (ref)	-				
Ex-smoker	0.899 (0.798, 1.013)	0.081	0.986 (0.859, 1.131)	0.838	1.128 (0.440, 2.892)	0.803
Current-smoker	0.936 (0.812, 1.080)	0.367	1.006 (0.864, 1.172)	0.934	1.005 (0.514, 1.963)	0.989
Physical activity	0.947 (0.841, 1.067)	0.372	0.948 (0.822, 1.095)	0.469	0.943 (0.761, 1.170)	0.596
MET-hours (hour/day)	-0.502 (-0.873, -0.132)	0.008	-0.492 (-0.972, -0.011)	0.045	-0.392 (-0.938, 0.154)	0.159
Adult height (cm)	-0.731 (-1.185, -0.277)	0.002	-0.242 (-0.624, 0.140)	0.213	-0.073 (-0.615, 0.469)	0.792
Medication use						
Anti-diabetic medications	1.076 (0.893, 1.296)	0.441	1.132 (0.918, 1.395)	0.246	1.002 (0.666, 1.508)	0.992
Anti-hypertensive medications	1.087 (0.956, 1.237)	0.204	1.131 (0.970, 1.320)	0.117	1.012 (0.796, 1.285)	0.925
Anti-dyslipidemic medications	0.891 (0.695, 1.143)	0.363	0.896 (0.642, 1.251)	0.519	0.823 (0.561, 1.207)	0.318

SD, Standard deviation; OR, Odds ratio; CI, Confidence intervals; USD, US dollars; MET, metabolic equivalent.

[1] The unweighted genetic risk score was constructed by summation of the number of risk alleles in *ALDH2* rs671 (G allele) an *ADH1B* in rs1229984 (C allele).

[2] Values are percentages (%) for categorical variables (e.g. *ALDH2* rs671 and *ADH1B* rs1229984), or means ± SD for continuous variables (e.g. genetic risk score).

[3] Values were derived by logistic regression for the categorical variables (Odds ratio [95% Confidence Interval]) or by linear regression for the continuous variables (beta coefficient [95% Confidence Intervals]) and represent the change in each variable by increase of genetic risk score.

Supplementary Table 5. Instrumental variable estimates of alcohol intake (g/day) and hypertension based on genetic risk score[1] for alcohol flushing.

	Total (n=2,011)		Men (n=1,468)		Women (n=1,127)	
**Diseases**	**OR (95% CI)**[2]	**P-value**[3]	**OR (95% CI)**	**P-value**[3]	**OR (95% CI)**	**P-value**[3]
Hypertension	1.035 (0.999, 1.072)	0.058	1.034 (1.002, 1.066)	0.012	1.024 (0.793, 1.322)	0.857
**Blood pressure**	**Beta coefficient (95% CI)**	**P-value**	**Beta coefficient (95% CI)**	**P-value**	**Beta coefficient (95% CI)**	**P-value**
SBP (mmHg)	0.406 (0.146, 0.665)	0.002	0.386 (0.152, 0.620)	0.001	0.547 (-1.129, 2.224)	0.522
Adjusting treatment effect +10mmHg[4]	0.446 (0.165, 0.726)	0.002	0.435 (0.181, 0.689)	0.001	0.491 (-1.325, 2.307)	0.596
Adjusting treatment effect +15mmHg[4]	0.465 (0.169, 0.762)	0.002	0.459 (0.190, 0.729)	0.001	0.463 (-1.457, 2.383)	0.636
DBP (mmHg)	0.196 (0.042, 0.350)	0.013	0.166 (0.028, 0.304)	0.019	0.448 (-0.552, 1.447)	0.380
Adjusting treatment effect +5mmHg[4]	0.216 (0.054, 0.378)	0.009	0.190 (0.046, 0.335)	0.010	0.419 (-0.631, 1.470)	0.434
Adjusting treatment effect +10mmHg[4]	0.236 (0.059, 0.412)	0.009	0.215 (0.057, 0.373)	0.008	0.391 (-0.752, 1.534)	0.502

OR, odds ratio; CI, confidence interval; SBP, systolic blood pressure; DBP, diastolic blood pressure

[1] The unweighted genetic risk score was constructed by summation of the number of risk alleles in *ALDH2* rs671 (G allele) an *ADH1B* in rs1229984 (C allele).

[2] ORs and beta coefficients by instrumental variable (IV) estimation were obtained from IV regressions with a two-stage least squares estimation method (in logistic and linear regression models, respectively), using genetic risk score for alcohol flushing as an instrument for alcohol intake. To predict the amount of alcohol intake, 0 for genetic risk score was regarded as a reference value.

[3] P values were derived from IV regression analysis with adjustment for age, sex (for total subjects), income, MET-hour/day and smoking status.

[4] To adjust treatment effect on blood pressure, sensible constants were added to the observed blood pressure values of all subjects on treatment (see Methods).

Supplementary Table 6. A comparison of alcohol intake between those who were homozygous for the G allele of the rs671 SNP with and without flushing .

	Total (n=1,505)			Men (n=1,468)			Women (n=1,127)		
**Alcohol intake (g/day)**	**N**	**Beta coefficient (95% CI)** [1]	**P-value**[1]	**N**	**Beta coefficient (95% CI)** [1]	**P-value**[1]	**N**	**Beta coefficient (95% CI)** [1]	**P-value**[1]
rs671 GG genotype without flushing symptom	1,224	1.000 (ref)	-	808	1.000 (ref)	-	416	1.000 (ref)	-
rs671 GG genotype with flushing symptom	281	-6.840 (-9.904, 3.776)	1.3×10^{-5}	174	-8.668 (-13.036, -4.300)	1.1×10^{-4}	107	-1.830 (-3.564, -0.095)	0.039

CI, confidence interval.
[1]Beta coefficient and P-values were obtained from linear regression using a combination of rs671 genotype and flushing as an exposure.

Supplementary Table 7. Distribution of genotypes of the *ALDH2* rs671 and *ADH1B* rs1229984 SNPs according to flushing status.

	Non-flusher (n=1,330)			Flusher (n=681)		
**rs1229984**	**rs671[1]**			**rs671[1]**		
	**GG**	**GA**	**AA**	**GG**	**GA**	**AA**
**TT**	668 (54.6)	56 (53.9)	0 (0.0)	179 (63.7)	226 (59.0)	11 (64.7)
**TC**	477 (39.0)	40 (38.5)	2 (0.4)	93 (33.1)	134 (35.0)	5 (29.4)
**CC**	79 (6.5)	8 (7.7)	0 (0.0)	9 (3.2)	23 (6.0)	21 (5.9)

[1] Values are number of the participants and percentages (%).
